# Bioinformatics Analysis of Exercise-Related Biomarkers in Diabetes

**DOI:** 10.1155/2022/1273153

**Published:** 2022-06-29

**Authors:** Xiaoju Bao, Jingyue Qiu, Qin Xuan, Xinming Ye

**Affiliations:** ^1^School of Sports Science and Engineering, East China University of Science and Technology, 130 Meilong Road, Shanghai 200237, China; ^2^School of Physical Science and Engineering, East China University of Science and Technology, No. 130, Meilong Road, Xuhui District, Shanghai 200237, China

## Abstract

**Background:**

Exercise is a regular behavioral activity that not only helps to lose weight but also reduces the risk of cardiovascular and cerebrovascular diseases. Diabetes is a common disease that plagues human health. It is shown that regular exercise can improve the insulin sensitivity of diabetic patients and have an important function in adjuvant therapy.

**Methods:**

We downloaded the GSE101931 dataset from the Gene Expression Omnibus (GEO) database, 10 samples were obtained from the GSE101931 dataset, including 5 before exercise and 5 postexercise samples, and GEO2R was used to screen the differentially expressed genes (DEGs) exhibited by a heat map. Then, the enrichment analysis of DEGs in Gene Ontology (GO) function was analyzed by Metascape, and the Kyoto Encyclopedia of Genes and Genomes (KEGG) pathway of DEGs was also analyzed by gene set enrichment analysis (GSEA). Next, the protein-protein interaction (PPI) network maps were drawn, and the hub genes were identified through Metascape. Finally, the expressions of the hub genes in the dataset were analyzed.

**Results:**

Totally, 116 upregulated DEGs and 1017 downregulated DEGs were identified from these data. These DEGs were mainly enriched in the platelet-derived growth factor receptor signaling pathway and mRNA processing. Then, the GSEA analysis showed that 6 KEGG pathways were associated with postexercise prediabetic samples, namely, ABC transporters, focal adhesion, MAPK signaling pathway, prion diseases, melanogenesis, and gap junction. Afterward, three hub genes (HSPA8, STIP1, and HSPH1) were highly expressed after exercise through the box plot analysis.

**Conclusion:**

A myriad of research results confirms that there is a certain connection between exercise and diabetes, which provides a favorable basis for emerging exercise into the treatment of diabetic patients.

## 1. Background

Exercise is a behavioral activity involving physical strength and skills, which can be generally divided into two categories, aerobic exercise and anaerobic exercise [1, 2]. Aerobic exercise is the exercise in which oxygen inhaled is equal to the exhaled and comes to a physiological equilibrium state during exercise [3]. Relative to aerobic exercise, anaerobic exercise is a high-speed and violent exercise of muscles under hypoxia [4]. No matter the aerobic exercise or anaerobic exercise, exercise has brought multiple benefits to our human health. It can not only promote blood circulation, control weight, strengthen physical fitness, and speed up metabolism but also reduce the risks of various diseases [5]. In addition, some studies also report that moderate exercise has a good intervention and improvement effect on diseases, such as Alzheimer's disease [6], Parkinson's disease [7], anxiety, depression [8], and heart failure [9]. However, the specific regulatory mechanism of exercise on human diseases is still unclear, and further exploration is needed.

Diabetes is the most common endocrine and metabolic disease caused by absolute or insufficient insulin secretion [10]. The traditional diabetes treatment medications mainly focus on insulin secretion and sensitization, which brings adverse side effects to patients, resulting in drug treatment incompliance and failure. In addition to insulin and oral hypoglycemic agents, other treatments such as gene therapy and induction of *β*-cells regeneration have not been widely applied [11]. At present, exercise therapy has been widely recognized and applied in the treatment of diabetes and its complications along with other therapies [12].

In recent years, high-throughput sequencing [13] has been applied to the research on various bioinformatics, which can screen and identify key biomarkers of various diseases or cancers [14]. Microarray technology is an emerging molecular biology technology based on a high-throughput platform, which is widely used in molecular biology [15]. The Gene Expression Omnibus (GEO) online database, as a global public repository, can be used to analyze high-throughput microarrays and next-generation sequence functional genomics datasets [16]. Herein, we obtain the GSE101931 dataset from the GEO database. The dataset contains sample data information related to exercise, which lays the foundation for the next research.

Our main purpose is to explore the connection between exercise and diabetes. First, we download the GSE101931 dataset file in the GEO database for analysis, then use GEO2R to draw the differentially expressed genes (DEGs) distribution map of the pre and postexercise prediabetic samples in the dataset, and analyze the Gene Ontology (GO) function enriched by DEGs through Metascape. Then, based on gene set enrichment analysis (GSEA) and the Kyoto Encyclopedia of Genes and Genomes (KEGG) pathways related to exercise are analyzed. Immediately afterward, the protein-protein interaction (PPI) network of DEGs is drawn and the hub genes are identified. The final results will help us obtain new treatment targets for diabetes and better apply exercise to the treatment of diabetes.

## 2. Materials and Methods

### 2.1. Analysis of Public Data

The GEO database is a public functional genomics database, from which the GSE101931 gene expression profile (GPL10558, Illumina HumanHT-12 V4.0 expression beadchip) was downloaded and saved. GSE101931 contained 38 samples, of which we chose 5 prior to exercise prediabetic samples and 5 postexercise prediabetic samples. Based on these data, the next step was carried out.

### 2.2. Identification of DEGs

GEO2R (https://www.ncbi.nlm.nih.gov/geo/geo2r/) is a differential expression analysis software for web applications that recognizes DEGs of GEO series samples. We set fold change (FC) > 1, *P* < 0.01 as the screening criteria for upregulated DEGs and FC < 1, *P* < 0.01 as the screening criteria for downregulated DEGs.

### 2.3. GO Enrichment Analyses of DEGs

Metascape (https://metascape.org/) is a versatile gene function annotation analysis tool that applies bioinformatics methods in batch gene and protein analysis to get a more detailed understanding. To clarify the GO function of DEGs, DEGs were analyzed by the Metascape tool, and then, the relationship between the enriched GO terms was studied.

### 2.4. GSEA Analysis

GSEA got prepared to assess the distribution trend of genes in a predefined gene table ranked by phenotype correlation to judge its influence on phenotype. On this basis, the KEGG pathways related with postexercise prediabetic samples were studied and analyzed. When *P* < 0.05, the enrichment results in GSEA were significant in statistics.

### 2.5. PPI Network Construction Analysis

The PPI network of DEGs in the sample data was constructed by Metascape, and the hub genes were screened by Molecular Complex Detection (MCODE).

## 3. Results

### 3.1. Identification of DEGs

**Figure 1:**
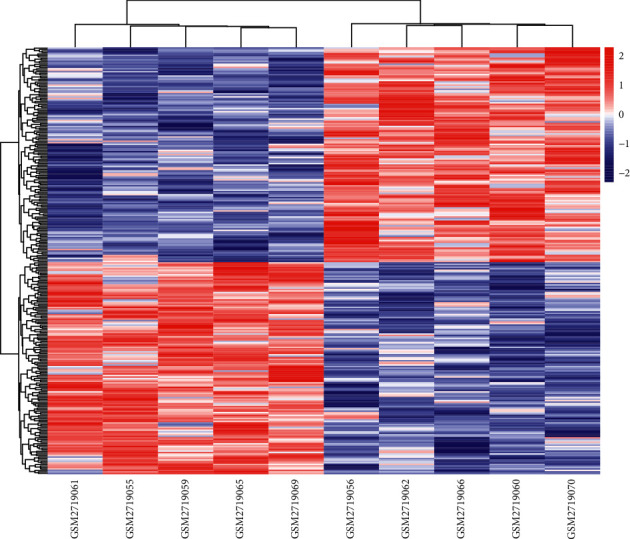
Distribution heat map of DEGs in the sample. Red represents the upregulation of DEGs, and blue represents the downregulation of DEGs.

Through the screening of 10 sample data by the criterion *P* value < 0.05 in the GSE101931 dataset, 1133 DEGs between 5 preexercise prediabetic samples and 5 postexercise prediabetic samples were identified, containing 116 upregulated DEGs and 1017 downregulated DEGs. The distribution heatmap of these DEGs in the data is shown in Figure 1.

### 3.2. GO Function Enrichment Analysis

**Figure 2:**
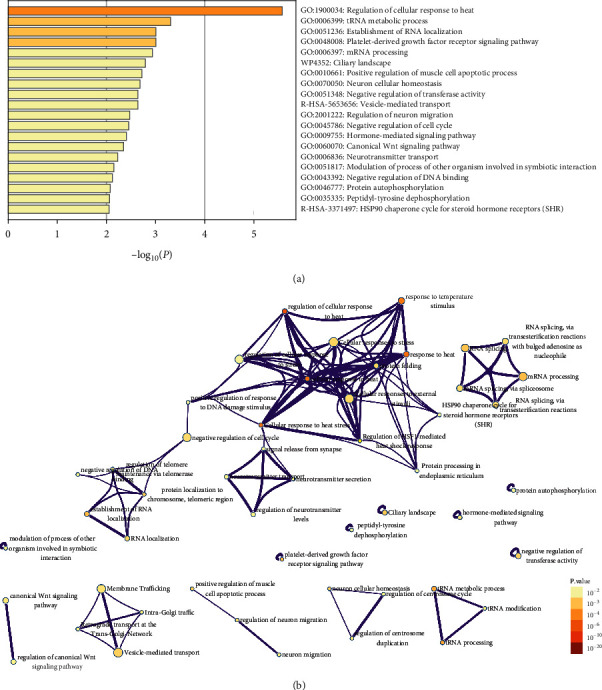
Functional clustering analysis of DEGs by Metascape. (a) GO function enriched by DEGs. (b) The network layout landscape transformed by the subset of representative items in GO function analysis.

GO analysis indicated that DEGs were mostly enriched in the regulation of cellular response to heat, tRNA metabolic process, establishment of RNA localization, platelet-derived growth factor receptor signaling pathway, and mRNA processing (Figure 2(a)).  Figure 2(b) is the network layout landscape transformed by the subset of representative items in GO function analysis. All the important items were classified and clustered into trees based on Kappa statistical similarity. Each item was shown by a circle node, and its size was proportional to the number of genes enriched by the item. The colors of different nodes represent different statistical significance.

### 3.3. GSEA analysis

**Figure 3:**
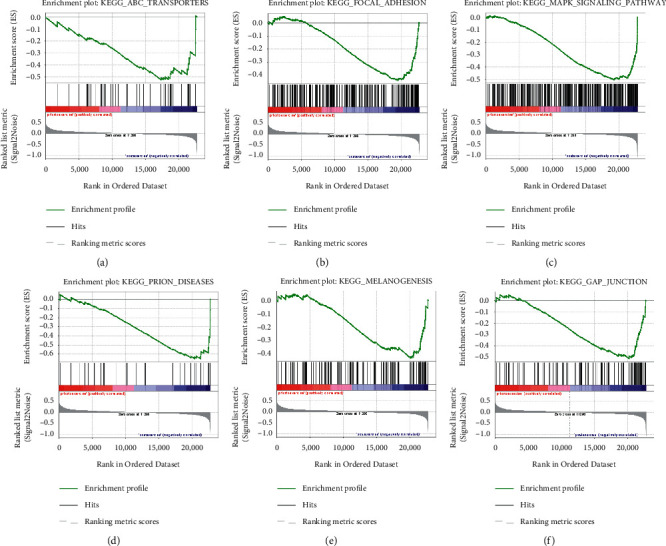
GSEA analysis showed KEGG pathways related with postexercise prediabetic samples. (a) ABC transporters. (b) Focal adhesion. (c) MAPK signaling pathway. (d) Prion disease. (e) Melanogenesis. (f) Gap junction.

Through GSEA to sort out the KEGG pathways related to postexercise prediabetic samples, we concluded that these genes were enriched in ABC transporters, focal adhesion, MAPK signaling pathway, prion diseases, melanogenesis, and gap junction (Figure 3).

### 3.4. PPI Network Construction and Module Analysis

**Figure 4:**
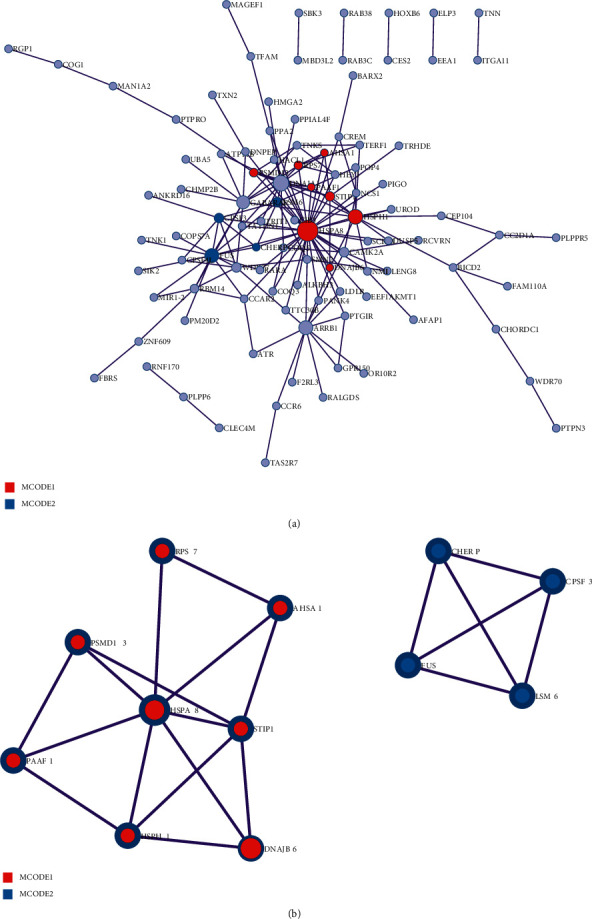
PPI network analysis by Metascape. (a) PPI network of DEGs. (b) MCODE was used to identify closely connected neighborhoods of proteins, and red and blue represent different MCODE.

A PPI network of 288 DEGs was constructed from the Metascape (Figure 4(a)), and the MCODE in the database was used for further analysis. Two modules were obtained according to the degree of interconnection among genes (Figure 4(b)). The red module contained 8 genes, namely, HSPA8, PSMD13, STIP1, AHSA1, RPS7, PAAF1, HSPH1, and DNAJB6. The GO functions enriched by these 8 genes were cellular responses to stress, cellular responses to external stimuli, and protein folding. The blue module contained 4 genes, CHERP, CPSF3, FUS, and LSM6. These four genes were significantly enriched in the mRNA splicing-major pathway, mRNA splicing, and processing of capped intron-containing pre-mRNA.

### 3.5. Analysis of Hub Gene Expression

**Figure 5:**
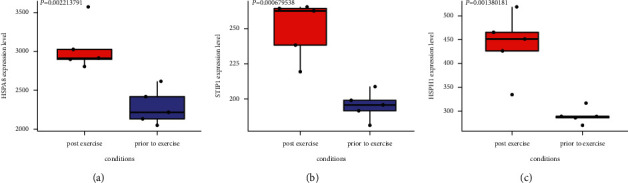
Box plot of the expression levels of 3 hub genes in the two sets of samples. (a) HSPA8. (b) STIP1. (c) HSPH1.

Subsequently, a box plot of the hub gene expressions in the dataset was drawn. Three hub genes were selected, namely, HSPA8, STIP1 and HSPH1, and their levels in postexercise and prior to exercise were compared. It could be seen from the results of Figures 5(a)–5(c) that the levels of HSPA8, STIP1, and HSPH1 in postexercise prediabetic samples were significantly higher than prior to exercise prediabetic samples.

## 4. Discussion

As we all know, proper exercise is beneficial to the body's physical and mental health. Studies have shown that exercise can improve the body's defense mechanism against diseases and the body's physiological functions [17]. Exercise not only plays a key role in arthritis [18] but also has good improvement effects on reducing cardiovascular and cerebrovascular diseases, inflammation, and high blood pressure [19]. The study by Han P et al. mentioned that exercise had a positive physiological and social psychological impact on patients after stroke [20]. The integration of myriad data confirms aerobic exercise as the chief form of cardiac rehabilitation [21]. It is very necessary for patients after stroke to properly perform aerobic exercises, strength training, flexibility exercises, and neuromuscular exercises in the later rehabilitation process. In addition, exercise also has a great effect on the treatment of mental illness. Knapen J et al. analyzed the impact of physical exercise on depressed patients and proposed that for patients with mild or moderate depression, exercise could achieve the same therapeutic effect as antidepressant drugs and psychotherapy. For patients with severe depression, exercise can also be used as a valuable supplementary treatment [22].

Diabetes is a metabolic disease characterized by high blood sugar, which mainly includes type 1 diabetes and type 2 diabetes [23]. The main manifestations are polydipsia, polyuria, polyphagia, weight loss, obesity, and fatigue [24]. The disease often causes complications, such as blindness, amputation, renal failure, and cardiovascular and cerebrovascular diseases [25]. According to statistics from the World Health Organization, the mortality rate of diabetes has been increasing in recent years, and it has become one of the top ten reasons for disease-related death globally [26]. The specific causes of diabetes have not yet been clarified, and different types of diabetes have different pathogenic factors. Moreover, clinically, a method has not been found to completely cure the disease, and the development of the disease can only be controlled through targeted treatments, such as some drugs or surgery [27]. In recent years, many studies have pointed out that exercise can be used in the treatment of diabetic patients. Matos M et al. conducted a study on this and found that exercise was an effective nondrug intervention for diabetic patients, and it had a good improvement effect on some complications [28]. The report by Lumb A et al. pointed out that aerobic exercise and anaerobic exercise could improve the blood sugar control of patients and were key components for the prevention and treatment of type 2 diabetes [29]. Therefore, exploring the mechanism of exercise regulating diabetes is helpful to the prognosis and treatment of diabetic patients.

We obtained the GSE101931 dataset from the GEO database. The dataset contained 10 data samples, 5 preexercise prediabetic samples and 5 postexercise prediabetic samples. Through GEO2R, 1133 DEGs were identified in these samples, 116 were upregulated DEGs and 1017 were downregulated DEGs. After analyzing the GO function of these DEGs, it was found that these DEGs were significantly enriched in the regulation of cellular response to heat, tRNA metabolic process, establishment of RNA localization, platelet-derived growth factor receptor signaling pathway, and mRNA processing. Harries LW et al. summarized the processing mechanism and mode of mRNA action in the mutation of single-gene diabetes and believed that mRNA processing had a certain potential to influence the complex etiology of diabetes [30]. Shen S et al. proposed that the platelet-derived growth factor regulated cell growth and division and had a special role in blood vessel formation. After research, it is concluded that the cellular mechanism of platelet-derived growth factor and its receptors in the pathogenesis of diabetes is regulated by signaling pathways, such as reactive oxygen species, ER stress, and NF-*κ*B [31].

GSEA was used to analyze the enriched KEGG pathway, and it was found that postexercise prediabetic samples were related with six pathways, such as ABC transporters, focal adhesion, MAPK signaling pathway, prion diseases, melanogenesis, and gap junction. The study by Koehn J et al. showed that ABC transporters were related to human diseases, such as familial high-density lipoprotein deficiency, retinopathy, diabetes, and cardiomyopathy, and were involved in a variety of biological processes in these diseases. It also listed 15 ABC transporters in human pancreatic *β* cells, confirming that some of the proteins were related to multidrug resistance in diabetes [32]. Focal adhesion, as a special site where intracellular integrin receptors interact with extracellular matrix and intracellular actin cytoskeleton, participates in the regulation of many diseases. Sun J et al. mentioned that the MAPK signal pathway could be used as a key regulator of various intracellular and extracellular signal transduction pathways, and abnormal phosphorylation would cause multiple diseases [33].

Based on the PPI network constructed by the online database Metascape, we identified three hub genes, namely, HSPA8, STIP1, and HSPH1. As a member of the heat shock protein 70 families, HSPA8 is usually related to auditory system disease and oral lichen planus. It is a molecular rheostat that regulates chaperone-mediated autophagy substrates in immune disorders. Zhang S et al. analyzed the expression and prognostic effects of STIP1 in human tumors and concluded that the high expression of STIP1 affected lymph node metastasis, clinical staging, and survival rate [34]. It can be used as a new index for the clinical treatment of tumors. HSPH1 is also a member of the heat shock protein 70 family and acts as an exchange factor with the nucleotides of HSC70. This gene plays a unique role as an inhibitory enzyme that inhibits the aggregation of misfolded proteins. At present, no one has studied the regulatory role of these three genes in severe diabetes. We suggest that exercise can regulate the expression of this hub gene, thereby inhibiting the occurrence and development of human diseases.

In short, to explore the relationship between exercise and diabetes, we screen the DEGs between 5 preexercise and 5 postexercise prediabetic samples in the GSE101931 dataset and identify 116 upregulated DEGs and 1017 downregulated DEGs. Then, through the GO analysis, mRNA processing and platelet-derived growth factor receptor signaling pathway are significantly related with exercise of prediabetic. In the GSEA analysis, ABC transporters are found significantly associated with postexercise prediabetic samples. Finally, HSPA8, STIP1, and HSPH1 are identified as hub genes through the PPI network, and their expression levels in the pre and postexercise prediabetic samples are compared. The expression levels of HSPA8, STIP1, and HSPH1 in postexercise prediabetic samples were significantly higher than prior to exercise prediabetic samples. The above results provide new research directions for the relationship between exercise and diabetes.

## Data Availability

The datasets used and/or analyzed during the current study are available from the corresponding author upon request.
